# Design of a Millimeter-Wave Radar Remote Monitoring System for the Elderly Living Alone Using WIFI Communication

**DOI:** 10.3390/s21237893

**Published:** 2021-11-26

**Authors:** Kai Guo, Chang Liu, Shasha Zhao, Jingxin Lu, Senhao Zhang, Hongbo Yang

**Affiliations:** 1 School of Biomedical Engineering (Suzhou), Division of Life Sciences and Medicine, University of Science and Technology of China, Hefei 230026, China; guok@sibet.ac.cn (K.G.); zsh1996@mail.ustc.edu.cn (S.Z.); 2 Suzhou Institute of Biomedical Engineering and Technology, Chinese Academy of Sciences, Suzhou 215163, China; zhaoss@sibet.ac.cn; 3 School of Mechanical and Electrical Engineering, Changchun University of Science and Technology, Changchun 130001, China; liuchang5224@163.com (C.L.); Q1246995415@163.com (J.L.)

**Keywords:** millimeter wave, phase unwrapping, FIR digital filter, health monitoring

## Abstract

In response to the current demand for the remote monitoring of older people living alone, a non-contact human vital signs monitoring system based on millimeter wave radar has gradually become the object of research. This paper mainly carried out research regarding the detection method to obtain human breathing and heartbeat signals using a frequency modulated continuous wave system. We completed a portable millimeter-wave radar module for wireless communication. The radar module was a small size and had a WIFI communication interface, so we only needed to provide a power cord for the radar module. The breathing and heartbeat signals were detected and separated by FIR digital filter and the wavelet transform method. By building a cloud computing framework, we realized remote and senseless monitoring of the vital signs for older people living alone. Experiments were also carried out to compare the performance difference between the system and the common contact detection system. The experimental results showed that the life parameter detection system based on the millimeter wave sensor has strong real-time performance and accuracy.

## 1. Introduction

Most existing measuring instruments require physical contact; they need to be attached to the patient for measurement and monitoring. Thus, these measuring instruments are not convenient for patients who require continuous monitoring for a long time. Moreover, in light of the current COVID-19 pandemic, non-contact vital signs monitoring equipment could become more important. Non-contact monitoring could help minimize the spread of the virus, and the use of non-contact monitoring methods could ensure the safety of health care personnel. Therefore, remote, non-contact health monitoring instruments are urgently needed [1].

As the elderly population continues to rise, the population model has gradually shown an “inverted pyramid” pattern. In view of the situation where there is no one to take care of the elderly living alone at home, monitoring the elderly and their daily routines will become particularly important [2]. The elderly population needs care, and their living conditions require real-time monitoring using professional equipment.

Therefore, there is a great social value to study indoor safety monitoring terminals that can detect the daily activity routines of the elderly and ensure the daily safety of the elderly living alone [3].

Millimeter wave is a new type of non-contact life signal detection method, which can detect the relevant life parameter signals produced by the human body due to heart and lung activities [4]. Compared to traditional ECG and pulse detectors, it has the characteristics of non-contact, and at the same time has a certain penetration ability, so it can be detected through obstacles such as clothing and quilts [5]. Compared to non-contact sensors of other systems such as infrared and laser, millimeter wave has the characteristics of low environmental interference, so it can be used in a domestic environment without general electromagnetic interference [6]. Due to its high frequency and small antenna size, millimeter wave sensors can construct a compact and cost-effective detection system [7].

With the above advantages, millimeter wave technology has huge application potential in medical diagnosis, health monitoring, disaster rescue, and other fields, and therefore has important research value and significance [8].

As early as the early 1970s, people had begun to study non-contact life detection technology. In 1971, Caro and Bloice began to study asphyxia using microwave measurements [3]. In 1975, Lin et al. developed an X-band detection device [9]. The device was based on single-frequency continuous wave, in which the radar emits electromagnetic waves and meets the object to form an echo. The receiver received the radar echo and demodulated the echo signal to obtain phase and frequency related information. Then, the signal processing algorithm was used to extract the relevant components of human heart and lung signals, so as to test the target’s physical signs [10]. Since the 21st century, with the development of microwave radio frequency and semiconductor technology, radar detection technology has received great attention in the field of physiological detection. In 2004, the Droitcour research team in the United States designed a biologic radar that could accurately detect cardiopulmonary information abnormalities within a 2m range [11]. Li’s research team at the University of Florida developed a c-band portable vital signs detector in 2007, with a transmission power of only 201xW and a detection accuracy of 80% within a distance of 2.8 m [12]. In 2009, 5.8 ghz continuous wave radar was developed, which can detect infant signs within 1.15 m [13]. In 2010, the company developed a 5.8 ghz radar chip with a 0.13 micron CMOS process and an adjustable bandwidth of over 1GHz. In 2008, Zito’s research team from Italy carried out research on non-contact detection devices based on ultra-wideband radar and successfully developed a set of cardiopulmonary signal detection devices, which passed the test in 2011. The accuracy of data obtained was similar to that of traditional physiological parameter detectors [14]. In 2009, Gupta’s team in Italy successfully developed a non-contact physiological parameter detection device based on a frequency modulation UWB radar, and in 2011, heart rate detection was successfully implemented. In 2015, RenL et al., using the frequency step continuous wave (Stepped Frequency Continuous Wave SFCW) radar, detected human body signs under different angles of breathing heartbeat detection accuracy [15]. Due to the interference of respiratory harmonics, environmental noise and the jitter of the target itself, the accuracy of the early non-contact sign detection is low. With the gradual development of research, some new methods have been proposed. In 2010, Khan et al. proposed a method to suppress respiratory harmonics using the MTD method. In 2017, the team also used Kalman filter and n-iteration method to effectively improve the stability and accuracy of heartbeat frequency detection. In 2011, Tariq et al. used wavelet transform to accurately extract breath signals [16]. In 2014, Nguyen et al. extracted heartbeat signals by using the periodicity of frequency distribution of respiratory harmonics without suppressing respiratory harmonics [17]. In 2016, Ren et al. demodulated the received signal through complex signal demodulation (CSD) and arctangent demodulation (AD). Then, they used the state-space model to obtain vital signs information from the signal phase information [18].

In the early days of the People’s Republic of China, there were few reports on the detection of vital signs by radar sensors, and application direction focused on earthquake and mine search and rescue. The fourth Military Medical University was the first to study biological radar technology, and the research team began the exploration of biological radar vital signs detection in 1998. In 2004, UWB based biologic radar was developed, and “Radar sign Detector” was developed for penetrating wall detection. In 2009, the Chinese Academy of Sciences developed the vital signs detection system using UWB radar, which can obtain relatively accurate signs in terms of detection algorithm. In 2014, Wang et al. used differential and cross multiply (DACM) algorithm to effectively solve the problem of arctangent demodulation phase interruption, thus improving the stability of physical sign detection. In 2015, Wu et al. proposed a spectrum-weighted accumulative method to improve the SNR of received signals. In 2018, Liang et al. proposed a vital sign monitoring system based on UWB radar, which utilizes the short-time Fourier Transform (STFT), Additionally, through the collection of empirical mode decomposition (EEMD Ensemble Empirical Mode Decomposition) spectrum to detect vital signs parameters, the method to solve the disadvantages of the EMD method, improved the detection accuracy [18].

This paper mainly studies the detection and separation methods of human breathing and heartbeat signals under the FM continuous-wave system [19].

The software and hardware design of the millimeter wave sensor system was mainly completed, and the FIR digital filter and the wavelet transform method were used to detect and separate breathing and heartbeat signals. On this basis, a method of suppressing respiratory harmonics based on adaptive filters was proposed for the interference of respiratory harmonics in the heartbeat signal. Through the transplantation of algorithms and the construction of software systems, we used cloud services to realize remote online calculation of vital signs parameters. With the smaller size of the radar module, vital signs monitoring can be realized without touch.

## 2. Materials and Methods

### 2.1. Theoretical Basis of Vital Parameter Detection Subsection

Breathing can cause chest wall displacement; the displacement was about 1~12 mm, and the breathing frequency range was 0.1~0.5 Hz; the heartbeat can cause the chest wall displacement to change 1~2 mm, and the heartbeat frequency range was 0.8~2.5 Hz [20] (see Table 1).

Breathing and heartbeat movements can cause tiny vibrations in the chest wall, and millimeter wave radar is able to detect this tiny displacement through the phase change of the signal [21]. So it can be used for predicting respiratory rate and heart rate [20] (see Table 1).

Traditional medical testing methods used thoracic cavity palpation and observation methods to detect breathing, but subjects may intentionally change their breathing rate and pattern when they perceive the measurement. Therefore, the use of non-contact methods to measure breathing frequency has great practical value [22].

### 2.2. Millimeter Wave Ranging Principle

Millimeter wave is a special class of radar technology that uses short-wavelength electromagnetic waves. The electromagnetic wave signal emitted by the radar system is reflected by other objects in the emission path, and by capturing the reflected signal, the radar system can determine information such as the distance, speed, and angle of the object. Since the target echo distance information of the LFMCW system radar can be simply processed using the Fast Fourier Transform (FFT). This was the most widely used modulation method, and has been used for the most in-depth research on chirp continuous wave radar.

**Figure 1:**
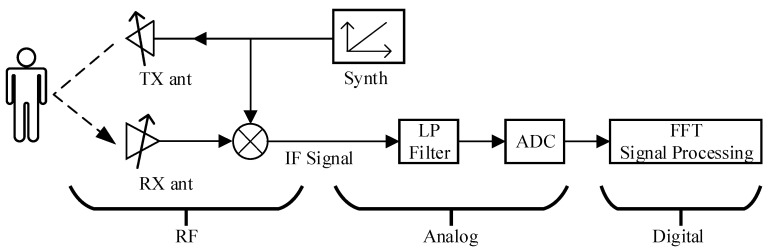
A simplified block diagram of the FMCW radar system [5,6,7,8,9].

The LFMCW radar system emits linear FM pulse signals and captures the signals reflected by objects in its emission path. Figure 1 is a simplified block diagram of the LFMCW radar system. The system working principle was as follows: first the signal source generated a linear FM pulse which was transmitted by the transmitting antenna; the object reflected and modulated a reflected linear FM pulse, captured by the receiving antenna; the mixer combined the transmit signal and the received signal together to generate an intermediate frequency (IF) signal [16].

The “mixer” was used to mix the receiving end (RX) and transmitting end (TX) signals to generate an intermediate frequency (IF) signal. The output of the mixer contained two kinds of signals, which were the sum and the difference between the Rx and Tx chirp frequencies. There was also a low-pass filter used to limit the signal, allowing only signals with a difference in frequency to pass.

The role of the mixer was to combine two input signals into a signal with a new frequency for two sinusoidal input signals $x_{1}$ and $x_{2}$:

(1) $$x_{1}=\sin\left(\omega_{1}t+\emptyset_{1}\right)$$

(2) $$x_{2}=\sin\left(\omega_{2}t+\emptyset_{2}\right)$$

The instantaneous frequency of the output signal $x_{out}$ was the difference of the instantaneous frequency of the two input signals, and the phase is equal to the difference of the phase of the two input signals:

(3) $$x_{out}=\sin\left[\left(\omega_{2}-\omega_{1}\right)t+\left(\emptyset_{2}-\emptyset_{1}\right)\right]$$

**Figure 2:**
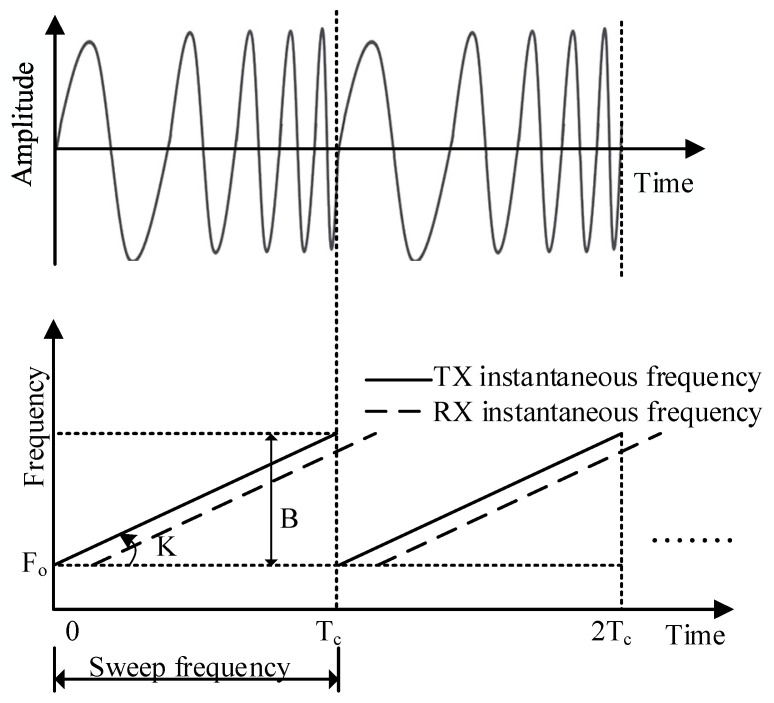
Detection of chest wall displacement [6,7,8,9,10,11].

In the FMCW radar system, the frequency of the emission signal increases linearly over time, and this type of signal was also known as the linear FM pulse signal. Figure 2 was a function of the amplitude of the linear FM pulse varying over time.

Figure 2 showed the radar waveform used for life signal detection in this paper. The radar waveform used was the sawtooth wave, $T_{c}$ was the sawtooth wave period, and B was the frequency modulation bandwidth. Within a sawtooth wave period, the radar signal emitted could be expressed as:

(4) $$S_{Tx}\left(t\right)=\exp\left(j\left(2{\pi f}_{c}t+{\pi \gamma t}^{2}+\varphi \right)\right)$$

In Formulas (2)–(4), $f_{c}$ was the center frequency of the radar transmitted signal, γ was the slope of sawtooth wave, and φ was the initial phase of the transmitted signal. The echo reflected after the radar signal irradiated on the chest wall of a human body can be expressed as:

(5) $$S_{Rx}\left(t\right)={\sigma S}_{Tx}\left(t-\frac{2R_{1}\left(\tau \right)}{C}\right)$$

In Formulas (2)–(5), C represented the speed of light and σ was the echo signal amplitude, a value related to the distance from the target to the radar and the radar reflection cross-sectional area (RCS), which was inversely proportional to the distance from the target to the radar and inversely proportional to the RCS. The RCS value of radar was related to the shape, size, material and other factors of the measured target. $2R_{1}\left(\tau \right)$/*C* is the delay of radar echo signal, the mathematical expression of $R_{1}\left(\tau \right)$ is:

(6) $$R_{1}\left(\tau \right)=d_{0}+R\left(\tau \right)$$

In Formulas (2)–(6), $d_{0}$ was the distance from the radar antenna to the target pleural motion center, and τ was the slow time increasing with the repetitions of the sawtooth wave. Radar echo signal was mixed with local oscillator signal to obtain an intermediate frequency signal:

(7) $$S_{IF}\left(t\right)=S_{Tx}\left(t\right)\times S_{Rx}^{*}\left(t\right)\;=\sigma \;exp\left(j\left(\frac{4\pi \gamma R_{1}\left(\tau \right)t}{C}+\frac{4\pi f_{c}R_{1}\left(\tau \right)}{C}-\frac{4\pi \gamma R_{1}^{2}\left(\tau \right)}{C^{2}}\right)\right)$$

(8) $$f_{b}=\frac{2\gamma R_{1}\left(\tau \right)}{C}$$

(9) $$\varphi_{b}=\frac{4\pi f_{c}R_{1}\left(\tau \right)}{C}-\frac{4\pi \gamma R_{1}^{2}\left(\tau \right)}{C^{2}}$$

According to Formulas (2)–(8), the frequency of the intermediate frequency signal was $f_{b}$, and the phase was $\varphi_{b}$. Since the value of $4{\pi \gamma R}_{1}^{2}\left(\tau \right)\ll C^{2}$, $4{\pi \gamma R}_{1}^{2}\left(\tau \right)/C^{2}$, was very small and can be ignored. Therefore, the phase $\varphi_{b}$ of the intermediate frequency signal could be further simplified as

(10) $$\varphi_{b}=\frac{4\pi f_{c}R_{1}\left(\tau \right)}{C}$$

According to Equations (2)–(8) and (2)–(10), it could be seen that both frequency $f_{b}$ and phase $\varphi_{b}$ of radar intermediate frequency signal $S_{IF}\left(t\right)$ contain displacement information $R_{1}\left(\tau \right)$ of detecting target chest cavity. IF frequency $f_{b}$ could be obtained by FFT of radar IF signal, but the maximum frequency resolution that FFT can achieve at this time was $1/T_{c}$. Put into the formula, it can be shown that the maximum range resolution that FFT of radar IF signal can obtain was C/2B, because the chest displacement caused by respiration and heartbeat was very small. Therefore, it was difficult to obtain the chest displacement change of the measured target directly through the radar intermediate frequency signal frequency $f_{b}$.

Use radar intermediate-frequency signal measured target to get the chest cavity displacement change law of each of the sawtooth sampling points as a row of the matrix, you can get a matrix (m for sawtooth wave number, n sampling points of each tooth), Vital signals related to breathing and heartbeat rates can then be obtained.

### 2.3. Software and Hardware Design for the Millimeter-Wave System

#### 2.3.1. System Framework

**Figure 3:**
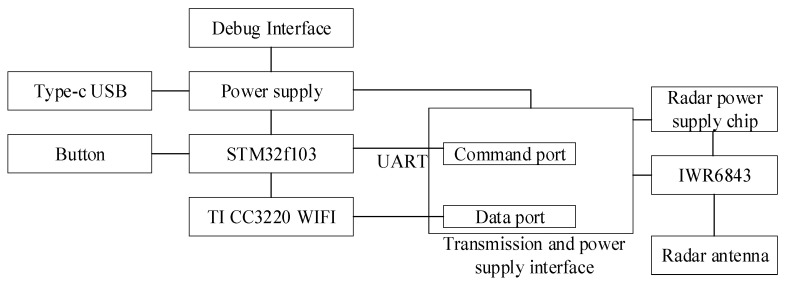
Hardware overall structure diagram.

The overall framework of the millimeter wave radar hardware is shown in Figure 3. The system constructed a complete modular circuit based on STM32f103 chip. The circuit mainly includes:

(1) Power supply module;
(2) TI C3220 WIFI communication module;
(3) IWR6843 mm-wave sensor;
(4) UART serial port interface for communication with the millimeter wave sensor module command system; TI CC3220 WIFI communicates with the millimeter wave sensor module data transmission interface to obtain raw data of the millimeter wave radar for back end data processing.

#### 2.3.2. Sending and Receiving Circuit

**Figure 4:**
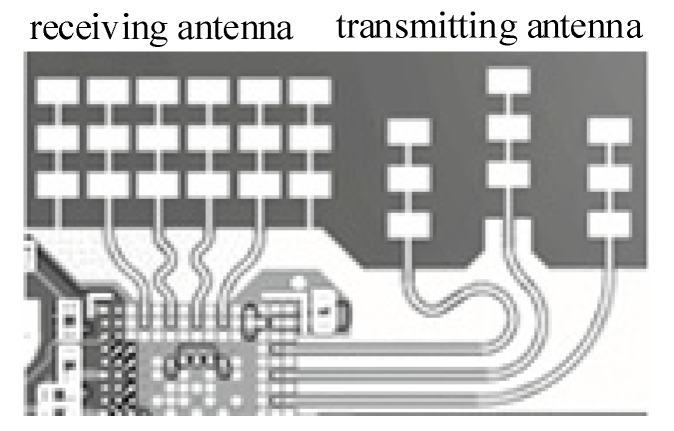
Antenna design [6,7,8,9,10,11].

The transceiver circuit was built based on the transceiver integrated chip IWR6843 of TI Company. The IWR6843 was an integrated single-chip mmwave sensor based on FMCW radar technology capable of operation in the 60-GHz to 64-GHz band. It was built with TI 45 nm RFCMOS process and enabled unprecedented levels of integration in an extremely small form factor. The IWR6843 was an ideal solution for low power, self-monitored, ultra-accurate radar systems in the industrial space. It had three transmitting channels and four receiving channels. The antenna design is shown in Figure 4.

**Figure 5:**
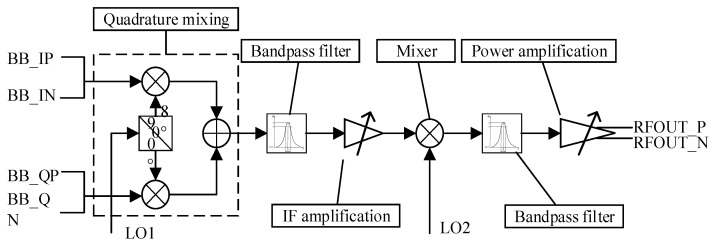
Schematic block diagram of the emission pathway [5,6,7,8,9,21].

The emission system consists of three parallel emission links, each with an independent binary phase as well as amplitude control (see Figure 5). The maximum output power for each emission link was 12 dBm, amplitude noise up to −145 dBc/Hz.

**Figure 6:**
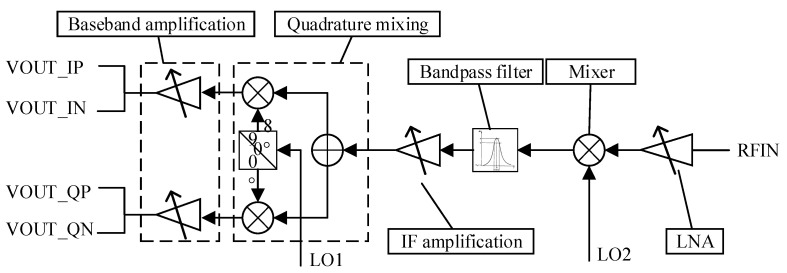
Block diagram of the receiving pathway principle [5,6,7,8,9,10,21].

The receiving system consists of four parallel channels, each including a low noise amplifier (LNA), mixer, intermediate frequency filter, and ADC (see Figure 6). All four receiving channels can be run simultaneously. Compared with the traditional mixer, the orthogonal mixing can effectively inhibit the mirror interference, while dividing the IF signals into real and virtual parts, thus reducing the ADC sampling rate. The receiver coefficient of noise was 15 dB, IF gain range of 24~48 dB, step 2dB, IF bandwidth of 5 MHz, the maximum sampling rate of 12.5 MHz.

The peripheral circuit included power management, external FLASH, high-speed interface, and other peripheral modules. The power management scheme was used to divide the input voltage into multiple channels by switching power supply, and further reduce the voltage by cascade low-voltage differential linear regulator (LDO). At the same time, π type filter was added between different levels to reduce noise. Finally, four outputs were formed, corresponding to the IO voltage of the main chip, the analog power supply voltage, the RF transceiver voltage and the core and SRAM voltage.

#### 2.3.3. Manufacturing the Hardware of the Radar System

**Figure 7:**
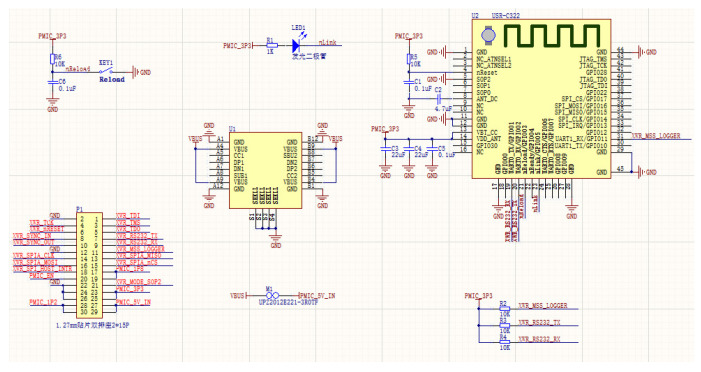
The schematic diagram of the WIFI communication circuit.

**Figure 8:**
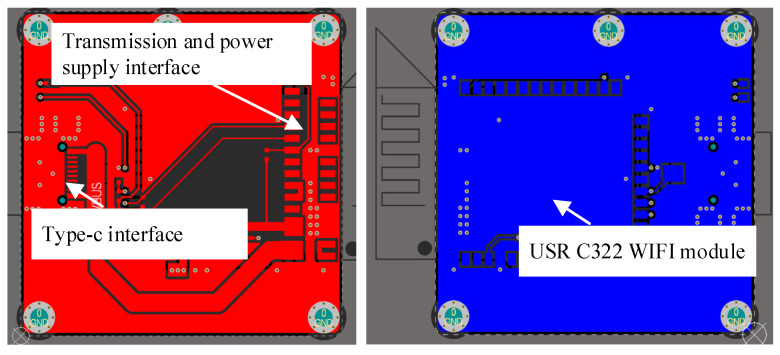
WiFi radar interface circuit.

We customized the radar core board from Changsha Ruigan Company, which provided the radar core board with customized interface based on our needs. We designed and welded the WIFI data board of the radar by ourselves, and there is no similar sample on the market. The final data transmission circuit board based on WIFI communication is shown in Figure 7 and Figure 8.

Figure 7 is the schematic diagram of the circuit, the WIFI circuit board and the radar board were connected by connecting pins, on which power supply and UART serial port transmission were integrated.

**Figure 9:**
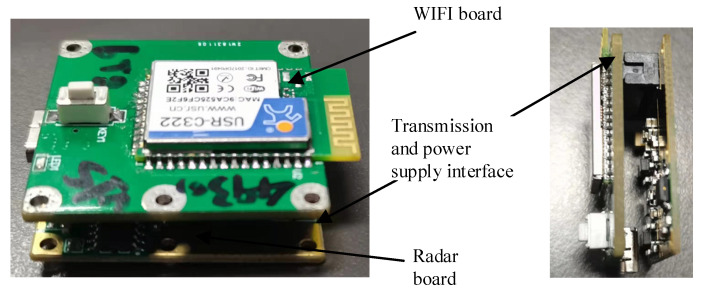
The Overall physical diagram of the hardware.

After we connect the core board and the Wi-Fi communication board we designed, the overall WIFI communication-based millimeter wave radar vital signs monitoring system is shown in Figure 9. The size of the whole hardware system was 34.0 × 32.0 × 10 mm.

The hardware mainly includes the core board and the WIFI data communication board. Firmware is an embedded program running on a circuit board, not software for algorithm calculations, so we generally treat firmware as part of the hardware. Two different hardware will have two different firmwares, the main contributions we made in the design and production are shown in Table 2.

From the above table, we mainly designed and produced the WIFI communication board, and the firmware of the two boards in the system was debugged, modified, and finalized by us.

#### 2.3.4. RF and Digital Front-End Configuration

In the LFMCW system, the frequency of the emission signal varies linearly over time. This periodic frequency scan was commonly referred to as chirp [23,24,25,26,27], as a linear FM pulse.

**Figure 10:**
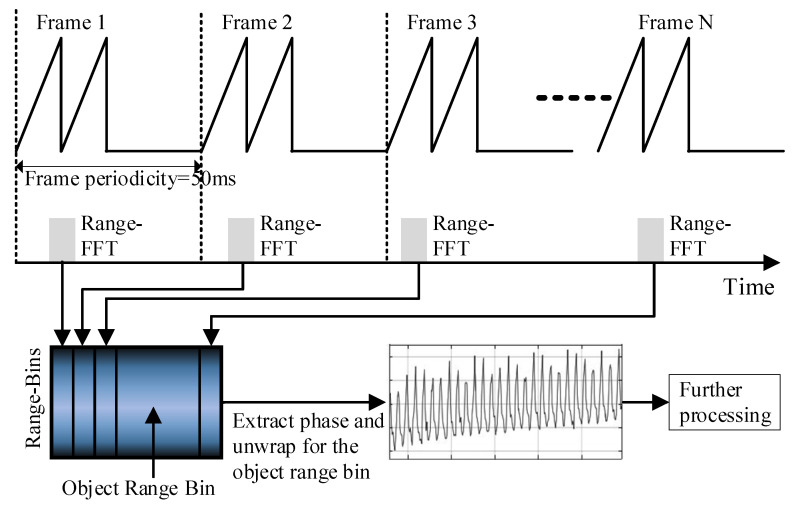
Emission waveform diagram [9,10,11].

The frame period was set to 50ms, and each frame contained two chirp structures per frame. During the data processing, the chirp of each frame was sampled, and then the FFT calculation of the distance dimension extracts the phase information of the distance unit where the object was being measured, which contains the chest wall vibration data, so reciprocated. Thus, for the original chest wall displacement data, the sampling rate was associated with the frame period, and the sampling rate of the original phase data was 20 Hz. In addition, according to the chip data manual, the gain range of the receiver was 24–48 dB, step 2 dB. Since this paper needs to detect minor shifts and has a low SNR, the intermediate frequency reception gain was set to a maximum of 48 dB. A schematic diagram of the emission waveform after the configuration is shown in Figure 10.

Sampling according to each frame cycle in the figure above, first ADC samples the median frequency signal according to the overall algorithm processing process, then distance dimension FFT processing, the target distance is calculated, and then a series of subsequent algorithm processing detection and separation of human respiratory and heartbeat signals [24,25,26,27].

#### 2.3.5. The Software Architecture and Design

**Figure 11:**
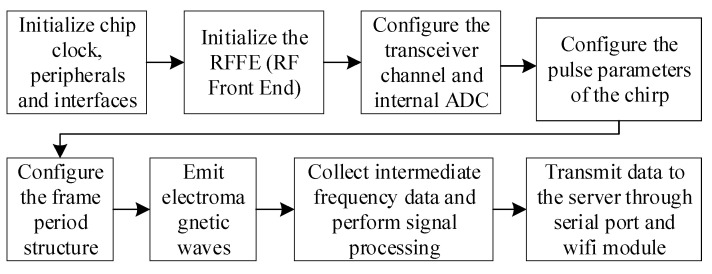
Software flow chart of millimeter wave radar module.

**Figure 12:**
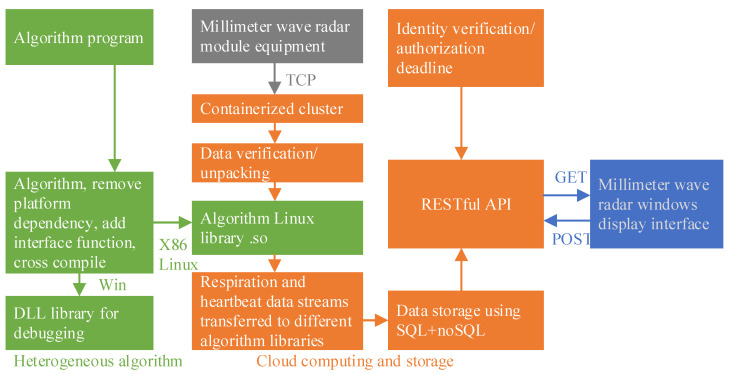
Software system architecture diagram based on cloud service.

The overall software workflow is shown in Figure 11, and the software system architecture based on cloud services is shown in Figure 12.

Our system used a cloud service software architecture. The device did not need to be directly connected to the computer via a data cable. The data was sent directly to the server via Wi-Fi, and the server’s program calculated the monitored data in real time by unpacking and computing the socket data. The data was saved on the server, and the software obtained and displayed the parameters in real time through POST and GET methods. The software system architecture diagram based on cloud service is shown in Figure 12. Our cloud server used Alibaba Cloud’s ECS server, and the server uses Ubuntu 18.04 system. The algorithm was transplanted and packaged into a dynamic link library of the Linux system (.so file). The netty framework was used on the server side to realize real-time data unpacking and algorithm calculation and store it in the database. The server used the RESTFUL framework of node.js, and the computer software obtained the calculated data through GET and post methods.

As shown in Figure 12, the algorithm must be run on a remote server for the system to implement cloud computing. Since the server system was Linux without an operation interface, we needed to transplant the algorithm. Considering the data was transmitted to the server through a socket, the socket framework used by the server was called netty, which was in java environment, so we need to package the algorithm of the C++ language. The C++ language algorithm needed to replace and rewrite the MFP-specific functions, and then define all functions as “extern ‘C’ int __declspec(dllexport)” so that the packaged. So, the file can be called externally. We needed to transfer the transplanted algorithm file into the computing server environment used and package the C++ language algorithm into the Linux platform dynamic link library by running the “g++ linuxDataCalculate.cpp -fPIC -shared -o libDataCal_1.so” command under bash.

After the algorithm was transplanted, it combined the netty framework to realize real-time unpacking and calculation of the data, and the result was stored in the server. The display software could obtain real-time data through the RestFUL API.

#### 2.3.6. The PC-Terminal Display Interface Design

The software functions designed based on this algorithm are roughly introduced as follows:

(1) Control the start, pause, and end of data collection and transmission functions;
(2) Real-time display of time-domain waveforms of breathing and heartbeat signals;
(3) Spectral analysis of breathing and heartbeat signals
(4) Estimate and display the respiratory rate and heart rate numerically.

**Figure 13:**
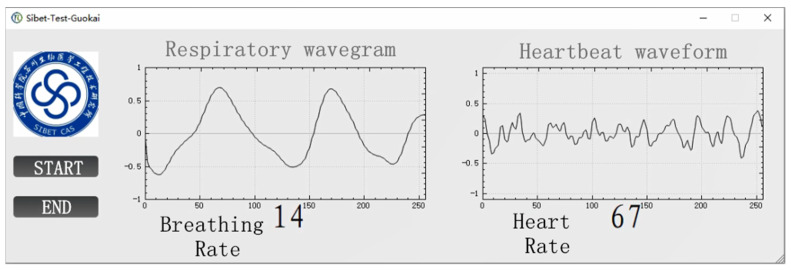
The PC-terminal display interface design.

The PC-terminal display interface design was shown in Figure 13.

In Section 2.4, the hardware and software design of the 60 GHz millimeter wave sensor was completed. The transceiver circuit was built based on the TI transceiver integrated chip IWR6843, and the transceiver system was composed of antennas. Design of peripheral circuits, including power supply, high-speed data interface, etc., constitutes a complete system. Then, the software configuration framework was designed according to the communication protocol format of the chip, and the RF and digital front-end configuration was carried out. The LFMCW sawtooth wave transmitting waveform is created, and the ADC sampling mode is configured to sample the front-end data, which makes preliminary preparation for the subsequent signal processing.

### 2.4. Physiological Parameter Detection Algorithm of Millimeter Wave Radar

**Figure 14:**
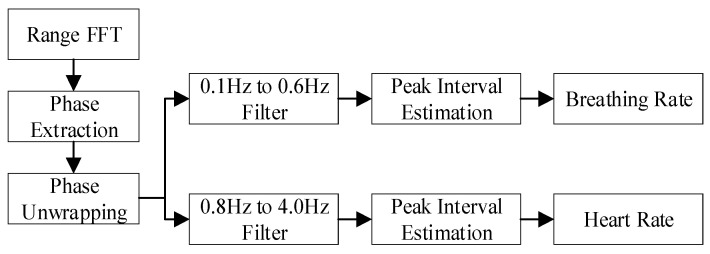
The two band pass filters were set to 0.1–0.6 Hz and 0.8–4.0 Hz corresponding to the respiratory and heartbeat bands, respectively, where the respiratory filter start frequency was 0.1 H z to filter out the interference of DC noise [9,10,11,12].

Figure 14 shows the systematic process of the detection of the human heartbeat and respiratory signals. According to the whole algorithm processing process, the middle frequency signal was first sampled by ADC, and then the range dimension FFT processing to calculate the target distance. Next, FFT calculation was performed for each chirp to find the distance unit corresponding to the target. Then, the phase information corresponding to the distance unit was extracted for subsequent algorithm processing.

(1) The two band pass filters were set to 0.1–0.6 Hz and 0.8–4.0 Hz corresponding to the respiratory and heartbeat bands, respectively, where the respiratory filter start frequency was 0.1 H z to filter out the interference of DC noise;
(2) Filters were designed using the fir1 function of order 41 and the window function selected a Hamming window;
(3) The raw data was filtered using the filter function.

The FIR filter can effectively isolate the breathing signal. Although it has some noise cancellation effect on the heartbeat signal, the heartbeat frequency information cannot be extracted from it, so the chest wall displacement signal will be processed by wavelet transform to isolate the heartbeat signal. Wavelet analysis was performed using a series of functions in MATLAB’s wavelet transformation toolkit, and the overall procedure was as follows:

(1) The appropriate wavelet basis function and decomposition layers are selected and one-dimensional discrete wavelet decomposition of the signal using the wavedec function;
(2) Use approximate coefficient and detail coefficient to extract the decomposition though appcoef and detcoef functions;
(3) The waverec function was reconstructed using the extracted approximation and detail coefficients to obtain the component sizes corresponding to the signal at different frequencies.

Section 2.4 briefly describes the signal detection and separation methods of human heartbeat and breathing. Section 3 will further verify the accuracy and stability of the system, and we conducted comparative experiments with traditional contact devices.

## 3. Results

### 3.1. Experimental Scene Setting

**Figure 15:**
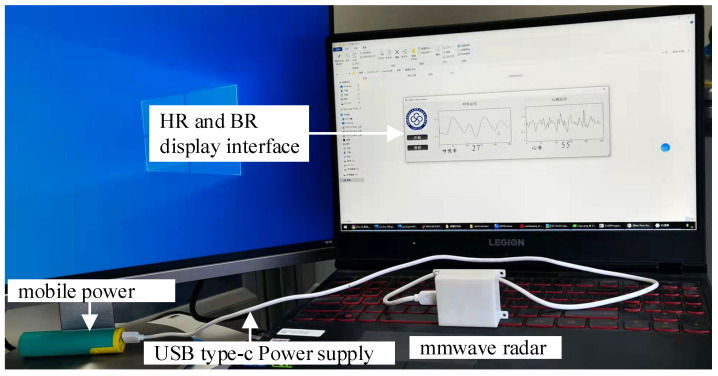
The millimeter-wave radar remote monitoring system for elderlies living alone based on WIFI communication.

This experiment was carried out in a laboratory environment, as shown in Figure 15. Subjects were two healthy women aged 28 years and 26 years old, measured under normal respiration. The subjects were within normal detection range, facing the millimeter-wave sensor and chest facing the sensor. Since this experiment was non-contact detection, the measured environment and human body’s movement were greatly disturbed, so it should be kept stationary as far as possible. There should be no metallic object between the sensor antenna and the subject.

**Figure 16:**
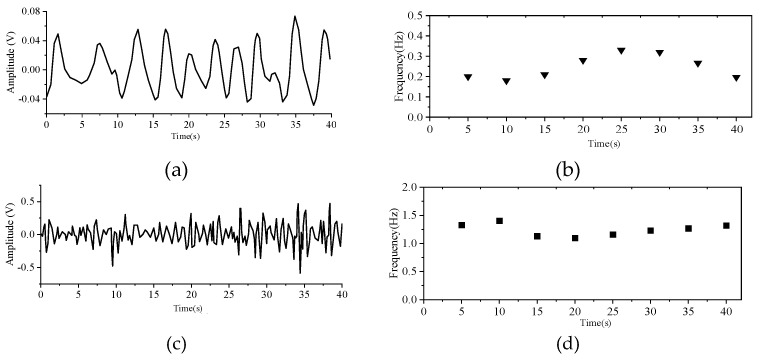
The experimental data. (**a**) Respiratory amplitude diagram, (**b**) Respiratory frequency domain analysis, (**c**) Heartbeat amplitude diagram, (**d**) Heartbeat frequency domain Value Hz.

The debugging program was downloaded to the IWR6843 through the software UniFlash provided by TI. The tester sat in front of the radar and remained as still as possible during the test. The radar test distance can reach about 2.5 m. Because the single-channel ECG module of the comparison device limited the measurement distance, the radar test distance is about 1 m. Figure 16 shows the measurements of the tester while maintaining normal breathing.

### 3.2. Experimental Comparison with Medical Grade Single-Lead ECG Equipment

**Figure 17:**
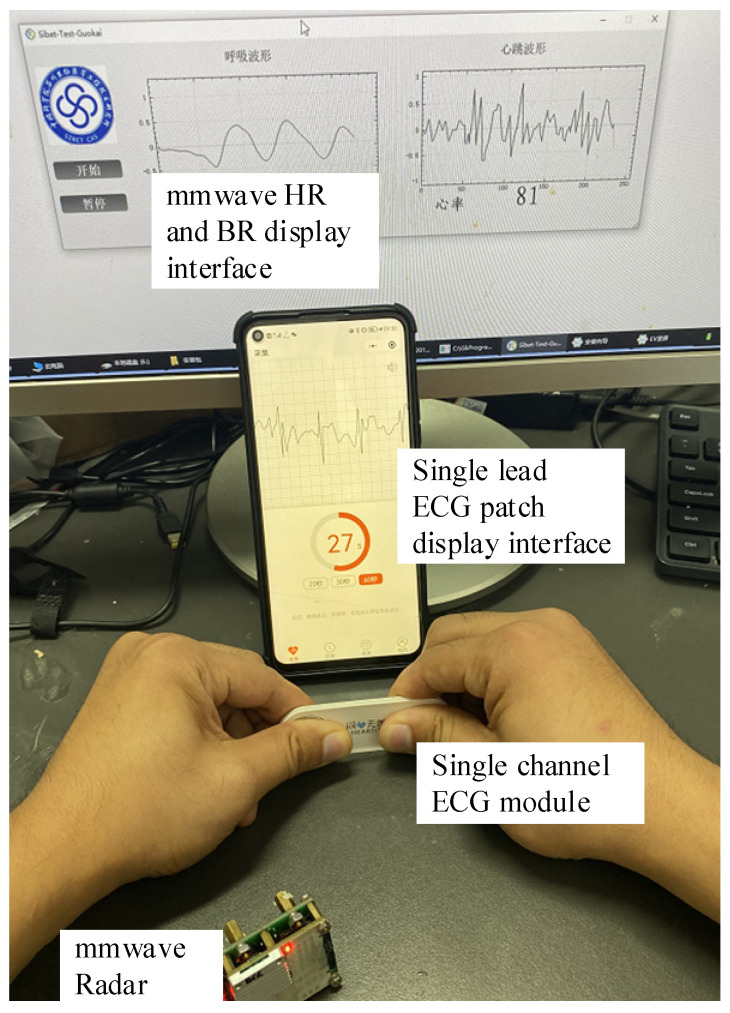
Comparative experiment of millimeter wave radar and single channel ECG module.

The previous article completed the separation of breathing and heartbeat signals and studied the influence of their harmonics. The relevant adaptive filtering algorithm has been proposed for simulation and experimental verification. Furthermore, to verify the system’s accuracy and stability, a comparative experiment was conducted with the conventional contact equipment. The selected contact device was Single channel ECG module. The investigation adopted the method of control variables to achieve synchronous detection. Two healthy subjects were measured in the normal breathing state, and two devices, millimeter wave sensor, and single channel ECG module were used for the experiment. Millimeter wave sensor will be detected by an algorithm based on the adaptive filter. A complete detection cycle was selected as a set of experimental data. In addition, respiration rate could not be detected in this experiment, so only heart rate data was compared. First of all, as shown in Figure 17, the comparison experiment was set. Subjects were asked to hold the electrodes on both sides of the single channel ECG module with both hands under normal breathing state to measure heart rate, and at the same time, heart rate was measured with the millimeter wave sensor.

**Figure 18:**
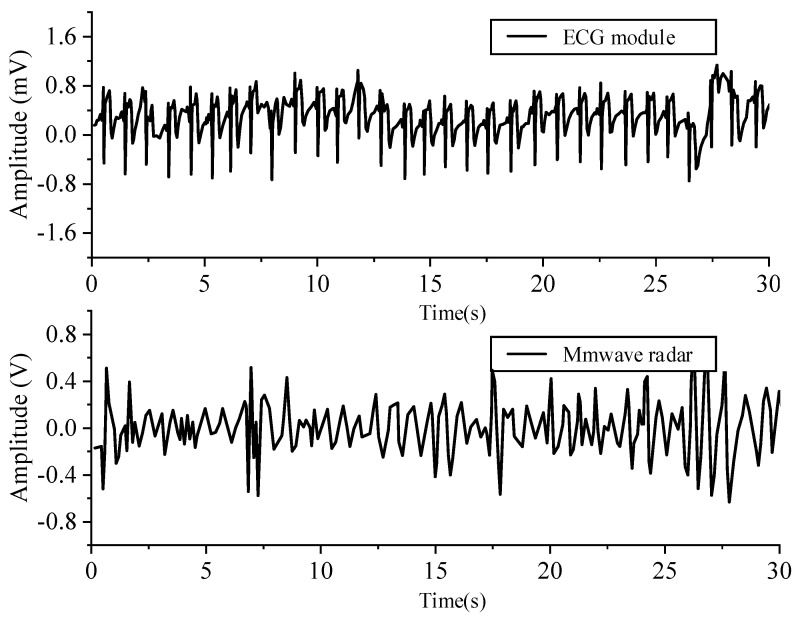
ECG and millimeter wave signals collected at the same time.

Then, through the comparative experiment of millimeter wave radar and single channel ECG module in Figure 18, ECG signal and heartbeat signal were collected simultaneously.

The figure shows the ECG and millimeter wave signals collected by the single lead ECG equipment. The heart rate calculated by millimeter wave radar was 63, and the heart rate calculated by medical single channel ECG device was 64.

**Figure 19:**
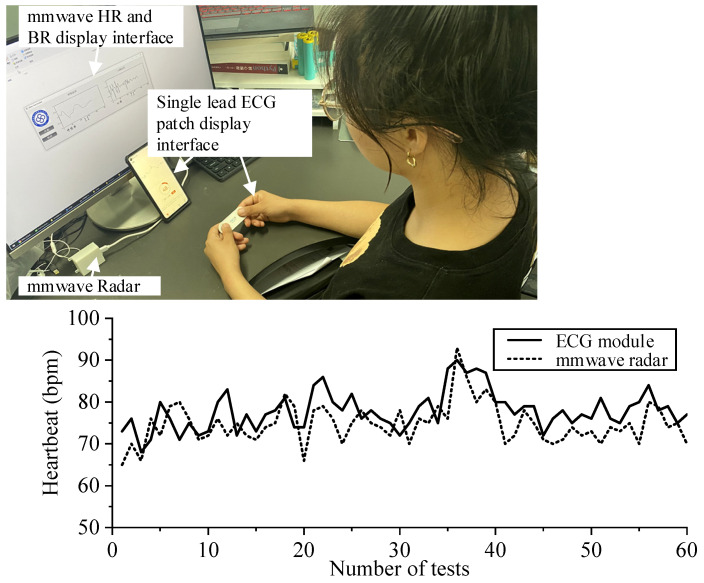
Tester 1.the comparative experiment of millimeter wave radar and single channel ECG module.

**Figure 20:**
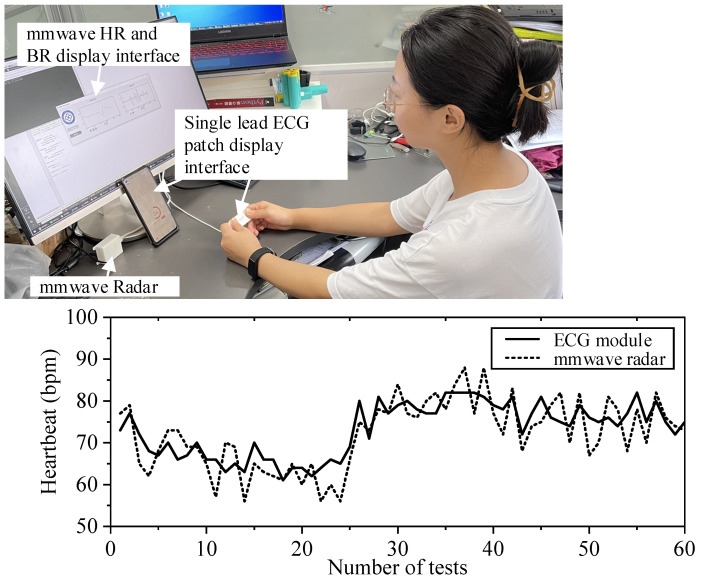
The comparative experiment of millimeter wave radar and single channel ECG module.

**Figure 21:**
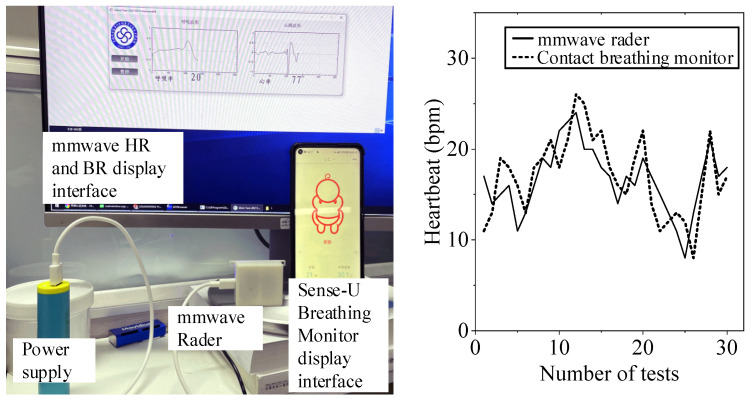
Comparative experiment with contact breathing monitor.

Continuous collection and testing 60 times of heart rate ratio; two comparative experiments (see Figure 19 and Figure 20):

We use a medical contact respiratory acquisition device called sense-u to conduct comparative experiments with millimeter wave radar. We recorded the respiratory rate values of the two devices every 30 s, collected 30 times, and drew the comparison curve. The experimental and comparative results are shown in Figure 21.

## 4. Discussion

As can be seen from the experimental results of tester 1 and tester 2 in FIG. 19 and FIG. 20, two healthy subjects were measured under normal breathing conditions, and millimeter-wave sensor and single channel ECG module were used for experiments simultaneously. The two heart rate data were overlapped and compared, and the error was within 6–7 bpm. You can see that the error fluctuation is slight. In the breathing comparison experiment in Figure 21, there was a certain gap between the contact breathing frequency and the non-contact breathing frequency, but the basic trend was the same. The measurement results of the millimeter wave sensor based on the adaptive filtering algorithm were close to those of the Single channel ECG module, which proved the measurement accuracy and robustness of the system in this paper.

## 5. Conclusions

This paper designed a millimeter-wave radar remote monitoring system for the elderly living alone, based on WIFI communication; a real-time and efficient life detection system. This paper conducted a software and hardware construction of a millimeter-wave sensor system and detected and separated it using an FIR digital filter and wavelet transform method for respiratory and heartbeat signals. The design of the system has good penetrating ability, can pass through obstacles such as clothes and bedding, is small in size, easy to integrate, and has higher efficiency. At the same time, combined with cloud computing, explore the possibility of remote vital signs monitoring by millimeter wave radar.

We hope to use our knowledge and technology and apply it to help disadvantaged groups in society. The system designed in this paper can realize real-time and efficient remote monitoring of vital signs (heart rate and breathing rate) of elderlies living alone, preventing them from having accidents without care or in guardianship loopholes.

## Figures and Tables

**Table 1:** Model of human life parameters [20,21,22].

Vital Signs Parameters	Amplitude	Frequency
Breathing signal	1–12 mm	0.1–0.5 Hz
Heartbeat signal	1–2 mm	0.8–2.5 Hz

**Table 2:** The main contributions we made in the hardware and firmware.

System Composition	Hardware Design and Manufacturing	Firmware of Board
The core board	Customized board from other company	Compile, debug, burn
The WIFI communication board	Design and manufacture	Debug, carry through

## Data Availability

The data presented in this study are available on request from the corresponding author.
